# Effects of Esketamine on Postoperative Cognitive Function in Elderly Patients Undergoing Pulmonary Lobectomy: A Randomised, Single-Blind Controlled Clinical Trial

**DOI:** 10.62641/aep.v53i6.2044

**Published:** 2025-12-17

**Authors:** Xueping Li, Huiting Mao, Jiao Luo, Weixiang Jiang, Peijuan Li, Leng Zhou, Long Tian, Zhenkun Liu, Yan Qiu

**Affiliations:** ^1^Department of Anesthesiology, West China Hospital, Sichuan University, 610041 Chengdu, Sichuan, China; ^2^West China School of Medicine, Sichuan University, 610041 Chengdu, Sichuan, China; ^3^Lung Cancer Center, West China Hospital, Sichuan University, 610041 Chengdu, Sichuan, China

**Keywords:** esketamine, postoperative cognitive dysfunction, pulmonary lobectomy, elderly patients

## Abstract

**Background:**

Elderly patients undergoing pulmonary lobectomy with incision are at a high risk for postoperative cognitive dysfunction (POCD). Intraoperative esketamine may offer potential neuroprotective benefits. This study aimed to evaluate the efficacy of intraoperative esketamine in reducing the incidence of POCD in elderly patients undergoing pulmonary lobectomy with incision.

**Methods:**

In this single-blind, controlled clinical trial, patients (aged 65–75 years) undergoing conventional pulmonary lobectomy were randomly allocated to receive esketamine (0.3 mg/kg/h) or remifentanil (0.1–0.2 μg/kg/min) during surgery. Cognitive function was assessed using the mini-mental state examination (MMSE) and negative emotional scores were recorded at baseline and multiple postoperative time points. Intraoperative and postoperative parameters, including heart rate (HR) and mean arterial pressure (MAP), pain scores and adverse events, were recorded. Blood samples were collected to measure amyloid-beta (Aβ) and microtubule-associated protein tau (tau) concentrations.

**Results:**

No significant difference was found in the incidence of postoperative delirium between the two groups, but the esketamine group exhibited a significantly lower incidence of POCD on days 1 and 3 postoperatively than the control group. The esketamine group also had significantly higher serum Aβ42/40 levels and significantly lower tau levels on day 1 postoperatively. At the end of surgery, the HR, MAP and pain visual analogue scale score of the control group were significantly higher than those of the esketamine group. No significant differences were observed in terms of adverse events between the two groups.

**Conclusion:**

Intraoperative administration of esketamine (0.3 mg/kg/h) was associated with a lower incidence of POCD and more stable hemodynamic indicators in elderly patients undergoing thoracic surgery, without increasing adverse events. The application of esketamine indicates a possible benefit with a favourable safety profile in reducing postoperative cognitive decline in this population.

**Trial Registration:**

Chinese Clinical Trial Registry, ChiCTR2200065266.

## Introduction

Postoperative cognitive dysfunction (POCD) including mild memory disturbances and some other forms of cognitive dysfunction is one of the most common complications in central nervous system complications after anaesthesia, significantly influencing prognosis and quality of life, especially amongst elderly patients [1]. Patients aged 60 years and above exhibit a significantly greater susceptibility to POCD following non-cardiac surgery and longer-lasting symptoms with an increased risk of death in the first year after surgery than their younger counterparts [2].

POCD is a common complication following thoracic surgery [3]. The lung is an important site of systemic inflammatory response, especially for pulmonary lobectomy [4]. Perioperative various factors, including surgery, mechanical ventilation and ischemia-reperfusion, can promote the release of cytokines in the lungs [4, 5]. These cytokines can enter the brain through blood circulation and damage the blood–brain barrier, triggering an inflammatory response, which could impair synaptic plasticity and cause neuron injury and further lead to memory decline and cognitive dysfunction [6, 7, 8].

With the increasing amount of lung surgeries, especially amongst elderly patients, optimising the anaesthesia plan to reduce the incidence of POCD is an urgent problem that needs to be addressed. Esketamine, a non-competitive N-methyl-D-aspartate receptor antagonist and an anaesthetic with good analgesic effect, has been increasingly recognised not only for its rapid antidepressant effects but also for its potential benefits in improving cognitive function in patients with depression [9, 10, 11]. Clinically, two randomised controlled trials have demonstrated that esketamine administration is associated with significant improvements in multiple cognitive domains, including executive function, attention and delayed recall [9, 12]. One study has indicated the potential neuroprotective effects of esketamine by preventing surgery-induced inflammatory responses and reducing neuronal damage in the hippocampus, indicating that these cognitive enhancements often occur independently of mood improvement, thus suggesting a direct neurocognitive benefit [13].

Previous studies have reported that continuous intraoperative infusion of esketamine during thoracic endoscopic surgery can remarkably reduce the use of opioids, maintain enhanced circulatory and respiratory stability and improve patients’ postoperative cognitive function [9, 14, 15]. However, current research on its use in long-duration conventional thoracotomy is limited. The longer duration of conventional thoracotomy than simple thoracoscopic surgery causes greater surgical trauma and more severe pulmonary inflammatory response, which can result in a higher incidence of POCD [16, 17]. Therefore, a randomised controlled single-blind trial was designed to investigate the effect of esketamine in elderly patients undergoing conventional pulmonary lobectomy with incision and preliminarily explore its potential mechanism.

## Materials and Methods

### Study Area

This prospective, single-centre, single-blind, randomised controlled clinical trial was conducted at West China Hospital of Sichuan University from August 2022 to October 2023. The study was approved by the Ethics Committee of Biomedical Research (Ethics approval number: 2022-1272), West China Hospital of Sichuan University. It was conducted in accordance with the Declaration of Helsinki. Informed consent was obtained from the patients and their families.

### Subjects

The inclusion criteria were as follows:

(1) Aged 65–75 years old.

(2) American Society of Anesthesiologists (ASA) physical status classification Ⅰ or Ⅱ.

(3) Preoperative diagnosis of advanced stage lung tumour (stages I–IIIA) requiring conventional pulmonary lobectomy with incision. 

(4) Without cognition dysfunction before surgery (baseline mini-mental state examination (MMSE) score of ≥24) [18].

(5) Have not received anaesthesia nor surgery in the past 6 months.

The exclusion criteria were as follows:

(1) History of organic disease, hypertension or arrhythmia and other organ disorders.

(2) History of psychiatric disease, intracranial occupation, intracranial aneurysm or intracranial haemorrhage in the past half year and patients with organic brain damage.

(3) History of glaucoma or high intraocular pressure and retinal detachment.

(4) Preoperative diagnosis of dementia or mild cognitive impairment or baseline MMSE ≤23 [18].

(5) Previously allergy to anaesthesia.

(6) Alcohol or illicit drug misuse disorder.

(7) Refusal to participate in the experiment or unable to understand the questionnaire due to low literacy level.

(8) Already involved in other clinical trials.

### Study Objectives

The primary objective was to investigate whether esketamine significantly reduces postoperative cognitive impairment in elderly patients undergoing conventional pulmonary lobectomy with incision. The secondary objective was to evaluate the analgesic effects of esketamine.

### Sample Size Calculation

According to the results of the authors’ previous pre-trial, the incidence of POCD in the group using esketamine was 5%, and that in the group without esketamine was 30%. Given a power of 80% and a significance level of 5%, a total of 70 patients were needed for the study. Considering the possibility of loss to follow-up or withdrawal from the trial, the final sample size was determined to be 94 cases. The sample size was based on the following formula:

$$n={\left[Z_{\alpha /2}\,\sqrt{\bar{P}\,\left(1-\bar{P}\right)}+Z_{\beta }\,\sqrt{P_{1}\,\left(1-P_{1}\right)+P_{2}\,\left(1-P_{2}\right)}\right]}^{2}/{\left(P_{1}-P_{2}\right)}^{2}$$

### Randomisation and Blinding

All subjects were from the Lung Cancer Center of West China Hospital. In accordance with the random number generated automatically by the computer, the subjects were divided into a control group (remifentanil group) with odd numbers and an experimental group (esketamine group) with even numbers. Although esketamine and remifentanil were colourless and transparent, blinding was implemented only for the participants and the data collection and analysis personnel. Esketamine and remifentanil were discontinuously infused 30 and 15 min, respectively, before the end of the surgery for esketamine’s longer duration of action, so one nurse who conducted the randomisation and the anaesthetists were unblinded to the group assignment.

### Study Design

On the day of surgery, a nurse prepared the test drug in accordance with patient grouping. The patients’ basic information and vital signs after entering the room were recorded (BeneVision N15, Shenzhen Mindray Bio-Medical Electronics Co., Ltd., Shenzhen, Guangdong, China) by the monitoring nurse during the operation, including heart rate (HR), peripheral capillary oxygen saturation (SPO_2_), blood pressure (BP, invasive BP monitored by arterial puncture under local anaesthesia), bispectral index (BIS; 115-043902-00, Shenzhen Mindray Bio-Medical Electronics Co., Ltd., Shenzhen, Guangdong, China) and body temperature. Anaesthesia was induced in all patients with midazolam (0.04 mg/kg; Jiangsu Nhwa Pharmaceutical Co., Ltd., Xuzhou, Jiangsu, China, Lot No.: TMZ24L31), propofol (1.5–2 mg/kg; Beijing Fresenius Kabi Pharmaceutical Co., Ltd., Beijing, China, Lot No.: 20220421), sufentanil (0.3–0.4 µg/kg; Yichang Humanwell Pharmaceutical Co., Ltd., Yichang, Hubei, China, Lot No.: AB4110212) and atracurium (2 mg/kg; Jiangsu Hengrui Pharmaceuticals Co., Ltd., Lianyungang, Jiangsu, China, Lot No.: C1C1212A). The two groups were accordingly administered with esketamine (0.3 mg/kg/h; Jiangsu Hengrui Pharmaceuticals Co., Ltd., Lianyungang, Jiangsu, China, Lot No.: 221014139) or remifentanil (0.1–0.2 µg/kg/min; Yichang Humanwell Pharmaceutical Co., Ltd., Yichang, Hubei, China, Lot No.: AD5030271), and both groups received sevoflurane (2–3%; Shanghai Hengrui Pharmaceutical Co., Ltd. Shanghai, China, Lot No.: 22060531) and intermittent administration of atracurium.

In both groups, BIS was maintained at 40–60 intraoperatively, and systolic BP was maintained at ±20% of the preoperative level by using metaraminol and ephedrine, with a mean arterial pressure (MAP) of 60 mmHg and above. Atropine was given if the HR was less than 50 beats/min. Insulation blankets were used to maintain the patients’ temperature at 36–37 °C. The patients’ basic perioperative information and intraoperative status were recorded.

Sufentanil (0.15 µg/kg) and ondansetron hydrochloride (Yichang Humanwell Pharmaceutical Co., Ltd., Yichang, Hubei, China, Lot No.: 221101A02) were given half an hour before the end of the operation, and remifentanil or esketamine was respectively stopped 15 min and half an hour before the end of the operation. After the operation, the tube was not removed until the patients awakened and resumed regular spontaneous respiration. Then, the patients were sent to the post-anaesthesia care unit (PACU), with vital signs being monitored continuously for at least half an hour and meeting the PACU standard before being returned to the ward. Intravenous analgesic pumps (100 mL; REHN(1), Jiangsu Renxian Medical Technology Co., Ltd., Nantong, Jiangsu, China) containing hydromorphone (10 mg; Yichang Humanwell Pharmaceutical Co., Ltd., Yichang, Hubei, China, Lot No.: AB40902111), dexmedetomidine (0.2 mg; Yangtze River Pharmaceutical Group Co., Ltd., Taizhou, Jiangsu, China, Lot No.: 22012026) and ondansetron hydrochloride (20 mg) were placed in all patients postoperatively without peripheral nerve block or local anaesthetic infiltration. If the visual analogue scale (VAS) score was ≥4 in PACU, additional boluses of three times at most were administered by pressing the analgesic pump. If the score remained more than 4 points, 5 µg of sufentanil was administered, and the rescue analgesic drug was included in the total postoperative opioid dosage. All patients were not allowed to receive sedatives, analgesics nor antiemetic drugs for the first 3 days after returning to the ward.

The criteria for termination of the research were as follows:

(1) The drug was not effective in maintaining anaesthesia on patients (e.g., severe BP fluctuations and increased BIS). (2) Patients experienced any serious adverse reactions during the trial. (3) Patients or family members asked to withdraw from the trial or had poor compliance and did not cooperate with the questionnaire.

### Primary Outcome

The primary outcome was the incidence of POCD as defined by patients’ MMSE scores, which were assessed at T0, T2, T3, T4 and T5. POCD was defined as a decrease of MMSE score by ≥3 points postoperatively compared with preoperatively [19, 20]. Time points were defined as follows: T0: the day before surgery, T1: the end of surgery, T2: 1 day postoperatively, T3: 3 days postoperatively, T4: 7 days postoperatively and T5: 1 month postoperatively.

### Secondary Outcomes

Some perioperative parameters, including HR; MAP at T0, T1 and T2; and pain VAS at T0, T1, T2 and T3, were monitored to provide a detailed assessment of the analgesic effect of esketamine. Hypotension was defined as a systolic BP decrease greater than 20% of baseline, and hypertension was defined as a systolic BP increase greater than 20% of baseline [21]. Psychiatric evaluation via confusion assessment method (CAM) was performed at T2, T3 and T4. CAM consists of four criteria: (1) acute onset and fluctuating course (self-report of confusion, disorientation or hallucinations, or observed fluctuations in consciousness, attention or speech); (2) inattention; (3) disorganised thinking and (4) altered level of consciousness. A patient was considered to have postoperative delirium (POD) if criteria 1 and 2 were met, along with either criterion 3 or 4 [22]. In addition, the patients’ anxiety-depression scores were assessed at T0, T2, T3, T4 and T5 in accordance with the hospital anxiety and depression scale (HADS), with its subcomponents for depression (HADS-depression subscale, HADS-D) and anxiety (HADS-anxiety subscale, HADS-A). Anxiety and depressive symptoms are defined as HADS-A and HADS-D scores greater than or equal to 8, respectively [9].

Blood samples were collected at T0, T2 and T3. Amyloid-beta 1-42 (Aβ42), amyloid-beta 1-40 (Aβ40) and tau concentrations were measured using enzyme-linked immunosorbent assay (ELISA) kits (Aβ42: DAB142, R&D Systems, Inc., Minneapolis, MN, USA; Aβ40: DAB140B, R&D Systems, Inc., Minneapolis, MN, USA; tau: ab269557, Abcam plc, Cambridge, UK). All assays were performed using the Varioskan LUX multifunctional microplate reader (Thermo Fisher Scientific Inc., Waltham, MA, USA).

### Statistical Analysis

SPSS software (version 26.0, IBM-SPSS Statistics, Chicago, IL, USA) was used for statistical analysis, with a *p* value < 0.05 (two-tailed) considered statistically significant. The normality of data distribution was assessed using Shapiro–Wilk test and Q-Q plot. All normally distributed data were presented as mean ± standard deviation and analysed using *t*-test or one-way analysis of variance (ANOVA). Non-normally distributed measures were expressed as median (interquartile range, IQR) and analysed using Mann–Whitney U test. Repeated-measure ANOVA was used to compare data at different time points within groups. Multiple comparisons were adjusted using a Sidak correction. Categorical variables were expressed as numbers (proportion, %). Chi-square test was used when the sample size N ≥40 and the theoretical frequency T ≥5. The corrected chi-square test was used when 1 ≤ T < 5. Fisher’s exact test was used when N <40 or T <1. Baseline imbalances between groups were corrected by analysis of covariance (ANCOVA) to assess adjusted between-group differences.

## Results

### Patient Characteristics

**Fig. 1.**
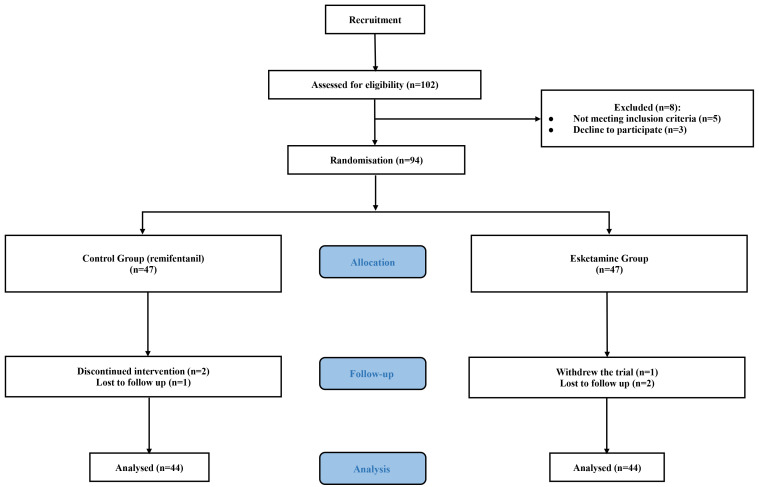
**CONSORT flow diagram for patients included in the study**.

At the beginning of the study, 102 patients were enrolled. A total of 88 patients, with 44 patients in each group, were ultimately analysed due to exclusion, withdrawal and loss to follow-up (Fig. 1). Age, gender, body mass index (BMI), educational level and smoking status were similar between the two groups (Table 1). The baseline MMSE, HADS-A and HADS-D scores showed no statistically significant differences between the groups (Table 1).

**Table 1.** **Baseline characteristics of participants between two groups**.

		Esketamine group (n = 44)	Control group (n = 44)	Statistic (Type/Value)	*p*-value
Age (years)		69 (67, 72)	69 (67, 73)	*Z* = –0.305	0.759
Gender n (%)				χ*²* = 1.359	0.244
	Male	39 (88.6%)	35 (79.5%)		
	Female	5 (11.4%)	9 (20.5%)		
BMI		24.66 ± 2.80	24.51 ± 3.62	*t* = 0.216	0.829
Educational level, n (%)				χ*²* = 0.873	0.350
	Junior high school and below	29 (65.9%)	33 (75.0%)		
	High school and above	15 (34.1%)	11 (25.0%)		
Smoking, n (%)				χ*²* = 1.620	0.445
	Current smoker	11 (25.0%)	16 (36.4%)		
	Never smoked	14 (31.8%)	10 (22.7%)		
	Former smoker	19 (43.2%)	18 (40.9%)		
HADS-A		3 (2, 3)	2 (1, 4)	*Z* = –1.063	0.288
HADS-D		5 (4, 6)	5 (3, 7)	*Z* = –0.819	0.413
MMSE		26 (24, 27)	26 (25, 27)	*Z* = –0.320	0.749

Note: All values are presented as mean ± standard deviation (SD) or median with interquartile range (IQR). BMI, body mass index; HADS-A, hospital anxiety and depression scale-anxiety subscale; HADS-D, hospital anxiety and depression scale-depression subscale; MMSE, mini-mental state examination.

### Intraoperative and Postoperative Patient Characteristics and Outcomes

**Fig. 2.**
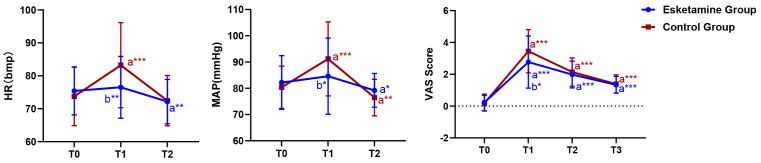
**Perioperative characteristics at different time points**. Note: All values are presented as mean ± SD. a* *p* < 0.05 versus the same group at T0, b* *p* < 0.05 versus the control group at the same time, a** *p* < 0.01 versus the same group at T0, b** *p* < 0.01 versus the control group at the same time, a*** *p* < 0.001 versus the same group at T0. T0: day before surgery, T1: end of surgery, T2: 1 day postoperatively, T3: 3 days postoperatively. HR, heart rate; MAP, mean arterial pressure; VAS, visual analogue scale.

The duration of anaesthesia (from the induction of anaesthesia to the patient leaving the operation room) was similar in two groups. We also compared the blood loss, crystalloid fluid infusion and length of hospital stay after surgery, and there was no significant difference between two groups. Hemodynamic measurements, including HR, MAP, and VAS score at the end of surgery, were higher in the control group than in the esketamine group (*p* < 0.05, Table 2). Compared with the preoperative levels, the HR and MAP of the control group significantly increased at the end of surgery (*p* < 0.05) and the MAP of the control group significantly decreased (*p* < 0.05) on day 1 postoperatively. Meanwhile, the HR and MAP of the esketamine group on day 1 postoperatively significantly decreased compared with the preoperative levels (*p* < 0.05, Fig. 2). Intergroup comparison showed that at the end of the surgery, the VAS score in the control group was significantly higher than that in the esketamine group, with no significant differences observed at the remaining time points. Similarly, the number of patients requiring sufentanil in PACU was far greater in the control group than in the esketamine group (*p* < 0.05, Table 2). Intragroup comparison showed that the VAS scores significantly increased from preoperative levels at the end of surgery and at days 1 and 3 postoperatively in both groups (*p* < 0.05, Fig. 2).

**Table 2.** **Comparison of perioperative characteristics and outcomes between two groups**.

		Esketamine group (n = 44)	Control group (n = 44)	Statistic (Type/Value)	*p*-value
Duration of anaesthesia (min)		218.5 (196.8, 246.3)	211.0 (187.3, 236.4)	*Z* = –1.382	0.167
Blood loss (mL)		140 (120, 180)	165 (123, 188)	*Z* = –1.272	0.203
Fluid infusion (mL)		350 (300, 350)	330 (330, 330)	*Z* = –0.695	0.487
Length of hospital stay after surgery (d)		6.3 (5.0, 7.9)	7.0 (5.5, 8.5)	*Z* = –1.009	0.313
Intraoperative dosage of sufentanil (µg)		35.0 (32.5, 40.0)	35.0 (32.5, 35.0)	*Z* = –1.439	0.150
Use of sufentanil in PACU n (%)		5 (11.4%)	13 (29.5%)	χ*²* = 4.470	0.034*
HR (bpm)					
	T0	75.4 ± 7.3	73.8 ± 8.9	*t* = 0.973	0.333
	T1	76.5 ± 9.4	83.2 ± 12.9	*t* = –2.975	0.006**
	T2	72.2 ± 6.8	72.5 ± 7.6	*t* = –0.177	0.860
MAP (mmHg)					
	T0	82.2 ± 10.2	80.4 ± 8.1	*t* = 0.927	0.357
	T1	84.6 ± 14.5	91.2 ± 14.1	*t* = –2.161	0.033*
	T2	79.2 ± 6.4	76.5 ± 6.9	*t* = 1.909	0.060
VAS score					
	T0	0.0 (0.0, 0.0)	0.0 (0.0, 0.0)	*Z* = –0.552	0.676
	T1	2.0 (2.0, 3.0)	3.0 (2.3, 5.0)	*Z* = –2.895	0.038*
	T2	2.0 (1.0, 2.0)	2.0 (1.0, 3.0)	*Z* = –0.973	0.397
	T3	1.0 (1.0, 2.0)	1.0 (1.0, 2.0)	*Z* = –0.290	0.701

Note: All values are presented as count (n) and percentage (%) or mean ± SD. *p < 0.05 versus the control group, **p < 0.01 versus the control group. T0: day before surgery, T1: end of surgery, T2: 1 day postoperatively, T3: 3 days postoperatively. PACU, post-anaesthesia care unit; HR, heart rate; MAP, mean arterial pressure; VAS, visual analogue scale.

### Incidence of POCD and POD Between Groups

No significant difference was found in the incidence of POCD at 7 days and 1 month postoperatively between the two groups. Notably, the incidence of POCD on days 1 (11.4% versus 31.8%, *p* < 0.05) and 3 (0.0% versus 13.6%, *p* < 0.05, Table 3) after the surgery were significantly lower in the esketamine group than in the control group. No significant differences were observed in the incidence of POD between the two groups (Table 3).

**Table 3.** **Incidence of POCD and POD between two groups**.

		Esketamine group (n = 44)	Control group (n = 44)	χ²	*p*-value
POCD incidence					
	T2, n (%)	5 (11.4%)	14 (31.8%)	5.437	0.020*
	T3, n (%)	0 (0.0%)	6 (13.6%)	4.472	0.034*
	T4, n (%)	0 (0.0%)	5 (11.4%)	3.393	0.065
	T5, n (%)	3 (6.8%)	6 (13.6%)	0.495	0.482
POD incidence					
	T2, n (%)	0 (0.0%)	1 (2.3%)	0.000	1.000
	T3, n (%)	0 (0.0%)	0 (0.0%)	0.000	1.000
	T4, n (%)	0 (0.0%)	0 (0.0%)	0.000	1.000

Note: All values are presented as count (n) and percentage (%). *p < 0.05 versus the control group. T2: 1 day postoperatively, T3: 3 days postoperatively, T4: 7 days postoperatively, T5: 1 month postoperatively, POCD, postoperative cognitive dysfunction; POD, postoperative delirium. POCD is defined as a decrease in MMSE score by ≥3 points postoperatively compared with preoperatively [19].

### Emotional Status

No significant differences were noted in the incidence of depression and anxiety between the two groups (Table 4).

**Table 4.** **Comparison of emotional status between two groups**.

		Esketamine group (n = 44)	Control group (n = 44)	Statistic (Type/Value)	*p*-value
Depression incidence					
	T0, n (%)	3 (6.8%)	7 (15.9%)	χ*² *= 1.805	0.179
	T2, n (%)	3 (6.8%)	2 (4.5%)	χ*² *= 0.000	1.000
	T3, n (%)	4 (9.1%)	9 (20.5%)	χ*² *= 2.256	0.133
	T4, n (%)	2 (4.5%)	4 (9.1%)	χ*² *= 0.179	0.672
	T5, n (%)	6 (13.6%)	7 (15.9%)	χ*² *= 0.090	0.764
Anxiety incidence					
	T0, n (%)	0 (0.0%)	2 (4.5%)	χ*² *= 0.512	0.474
	T2, n (%)	0 (0.0%)	0 (0.0%)	χ*² *= 0.000	1.000
	T3, n (%)	0 (0.0%)	1 (2.3%)	χ*² *= 0.000	1.000
	T4, n (%)	0 (0.0%)	0 (0.0%)	χ*² *= 0.000	1.000
	T5, n (%)	2 (4.5%)	4 (9.1%)	χ*² *= 0.179	0.672

Note: All values are given as mean ± SD. T0: day before surgery, T2: 1 day postoperatively, T3: 3 days postoperatively, T4: 7 days postoperatively, T5: 1 month postoperatively.

### Adverse Events After Surgery

No statistically significant differences were found in terms of the number of patients experiencing nausea, vomiting, dizziness, hypersomnia, hallucinations, hypotension, hypertension or respiratory depression (*p* > 0.05, Table 5).

**Table 5.** **Postoperative adverse events between two groups**.

	Esketamine group (n = 44)	Control group (n = 44)	χ²	*p*-value
Dizziness, n (%)	6 (13.6%)	3 (6.8%)	0.495	0.482
Nausea, n (%)	9 (20.5%)	4 (9.1%)	2.256	0.133
Vomiting, n (%)	5 (11.4%)	2 (4.5%)	0.621	0.431
Hypersomnia, n (%)	5 (11.4%)	3 (6.8%)	0.137	0.711
Hallucinations, n (%)	0 (0.0%)	0 (0.0%)	0.000	1.000
Hypotension, n (%)	2 (4.5%)	4 (9.1%)	0.179	0.672
Hypertension, n (%)	5 (11.4%)	3 (6.8%)	0.137	0.711
Respiratory depression, n (%)	2 (4.5%)	1 (2.3%)	0.000	1.000

Note: All values are presented as count (n) and percentage (%).

### Blood Biomarkers

**Fig. 3.**
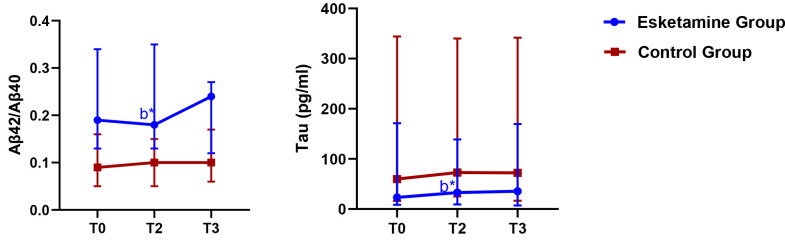
**Changes of perioperative biomarker level at different time points**. Note: All values are presented as median with IQR. b* *p* < 0.05 versus the control group at the same time. T0: day before surgery, T2: 1 day postoperatively, T3: 3 days postoperatively, Aβ, amyloid-beta; tau, microtubule-associated protein tau.

After adjusting for baseline differences by using ANCOVA, the esketamine group showed significantly higher Aβ42/40 levels than the control group on day 1 postoperatively (*p* < 0.05, Table 6). Notably, the Aβ42/40 levels were marginally higher in the esketamine group than in the control group on day 3 postoperatively, though the difference was not statistically significant (*p* = 0.067). The tau levels were significantly lower in the esketamine group than in the control group on the day after surgery (*p* < 0.05). Intragroup comparisons showed no significant differences in Aβ42/40 and tau concentrations on days 1 and 3 postoperatively compared with the preoperative levels (*p* > 0.05, Fig. 3).

**Table 6.** **Comparison of serum Aβ and tau concentrations between two groups**.

		Esketamine group (n = 24)	Control group (n = 25)	*p*-value
Aβ42/40				
	T0	0.19 (0.13, 0.34)	0.09 (0.05, 0.16)	0.001**
	T2	0.18 (0.13, 0.35)	0.10 (0.05, 0.15)	0.035^&^*
	T3	0.24 (0.12, 0.27)	0.10 (0.06, 0.17)	0.067^&^
Tau (pg/mL)				
	T0	23.10 (8.38, 171.31)	59.94 (16.25, 344.25)	0.112
	T2	32.93 (9.12, 138.75)	72.75 (25.21, 340.13)	0.038*
	T3	35.59 (7.16, 169.64)	72.20 (16.60, 341.94)	0.143

Some participants declined invasive blood sampling, and extreme outliers were excluded. The final sample size included in the analysis is shown in the table. The reported *p*-values for Aβ42/40 at T2 and T3 were derived from the results adjusted for baseline values by using analysis of covariance. All values are presented as mean ± SD or median with IQR. *p < 0.05 versus the control group, **p < 0.01 versus the control group, ^&^*p* values were corrected by analysis of covariance. T0: day before surgery, T2: 1 day postoperatively, T3: 3 days postoperatively, Aβ, amyloid-beta; tau, microtubule-associated protein tau.

## Discussion

This study showed that continuous intraoperative infusion of esketamine at a dose of 0.3 mg/kg/h may contribute to a reduction in the incidence of POCD, alongside favourable modulation of Aβ and tau, which are related to cognition.

Regarding the dosage selection for intraoperative administration of esketamine, existing studies have adopted varying dosage regimens, with most studies recommending a dose range of 0.25–0.5 mg/kg [9, 14, 23]. On the basis of the results of preliminary experiments and a balance between adequate analgesia and prevention of delayed recovery from anaesthesia, a continuous esketamine administration regimen at 0.3 mg/kg/h was ultimately adopted, which showed stable haemodynamics and favourable results.

POCD is a common complication after surgery, mainly manifested as a decrease in postoperative cognition [1]. The neuroinflammatory response to surgery is a major contributor to POCD by provoking prolonged microglial activation, synaptic impairment, reduced neurogenesis and programmed neuronal cell death [17]. Although the onset time of POCD is mainly in weeks to months after surgery, some research reported that the shortest time that patients were observed until they developed POCD was the first day of the first week and the longest time was 12 months [24, 25]. Amongst elderly patients undergoing elective surgeries, 41.1% were observed to have early POCD at discharge, whereas 12.7% showed late POCD 3 months later [2]. Previous research showed that esketamine could alleviate surgery-induced inflammatory responses [13]. The results of the present study found that the incidence of POCD was higher within the first 3 days only, but without significant differences at T4 and T5. This finding may be related to the patients’ age, limited administrated dosage of esketamine during surgery and its half-life of only 5 hours in the human body [10].

POD, a key risk factor for POCD, typically peaks within the first 3 days after surgery and involves different kinds of mental status, such as inattention, disorientation and consciousness disturbance [26]. However, esketamine could reduce the incidence of POCD significantly only on the first and third day after surgery, but it had no effect on POD. Unlike POCD, the pathogenesis of POD is primarily driven by acute cerebral dysfunction resulting from neurotransmitter imbalance, particularly dopamine excess and acetylcholine deficiency, often occurring in the context of predisposing factors and acute physiological stressors [12, 27]. This differential effect on POCD versus POD also precisely indicates that the protective mechanism of esketamine may be achieved by stimulating the remodelling of hippocampal neurons and improving the function of neurons that are related to POCD [24, 28], rather than by acting on the cholinergic system associated with POD [12].

The level of Aβ and tau in the cerebrospinal fluid (CSF) following surgery can indicate cognitive impairment [29]. Although S100β protein is a sensitive indicator reflecting early nerve damage, it has more predictive value for delirium [30]. Some studies [29, 31] have confirmed that patients with mild cognitive dysfunction exhibit a decrease in Aβ42/40 and an increase in tau, suggesting early nervous pathological changes. Barthélemy * et al*. [32] demonstrated that plasma tests of Aβ and tau performed as effectively as standard CSF tests. Thus, given the feasibility of the clinical trial and the minimisation of patients’ discomfort as much as possible, blood tests were utilised in the present study instead of CSF. The blood Aβ and tau concentrations in the first 3 days were tested to examine their association with early POCD. The control group exhibited significantly lower Aβ42/40 ratios and significantly higher tau levels on the first postoperative day only. Correlation analyses were conducted between the levels of the two biological proteins and the corresponding MMSE scores at respective time points. However, the analyses yielded low R-values that were not statistically significant due to the limited sample size. Therefore, the mechanism by which esketamine improves POCD may involve altering the expression of these two cognition-related biological proteins. Further studies with a larger sample size are needed to investigate the specific mechanism of action of esketamine and its relationship with these two proteins.

Large opioid dosage and abnormal perioperative cerebral perfusion are significant factors in the development of POCD [33, 34]. In the present study, the esketamine group exhibited more stable postoperative vital signs, along with significantly reduced VAS scores on day 1 postoperatively and a lower need for rescue sufentanil in PACU. These findings suggest the effective analgesic properties of esketamine and its potential to protect cognitive function by minimising perioperative opioid consumption and improving brain perfusion through maintaining stable circulation. The most common adverse events associated with esketamine include nausea, vomiting and dizziness [35]. Adverse events were observed in both groups. However, the incidence showed no significant difference between the two groups, indicating that the side effects of esketamine were not pronounced.

Luo* et al*. [9] reported that a single intraoperative injection of 0.5 mg/kg of esketamine can effectively relieve anxiety and depression in patients during the early postoperative period. However, the present study demonstrated no significant difference in the incidence of anxiety and depression between the two groups at various time points after surgery. This finding may result from the different administration methods and dosage regimens, potentially leading to differences in pharmacokinetic characteristics and peak concentrations [9].

This study has several limitations that warrant further discussion. Firstly, MMSE was employed as the primary tool for assessing cognitive function. Whilst MMSE is widely used, it has inherent limitations in detecting mild cognitive deficits that manifest in the early stages of a disorder. In addition, no uniform standard exists regarding the diagnosis of postoperative cognitive impairment, and different standards may lead to heterogeneity in the results. Secondly, only one dose of esketamine was studied, and the follow up was 1 month only. Future studies should consider a larger sample size and an extended follow-up duration to comprehensively evaluate the sustained impact of esketamine on POCD.

## Conclusion

This study demonstrates that intraoperative administration of esketamine (0.3 mg/kg/h) may reduce the incidence of POCD and improve hemodynamic stability in elderly patients undergoing conventional pulmonary lobectomy with incision. Future studies should explore the dose–response relationships, long-term cognitive outcomes and detailed mechanisms.

## Availability of Data and Materials

The data presented in this study are available upon request from the corresponding author.

## References

[1] Needham MJ, Webb CE, Bryden DC (2017). Postoperative cognitive dysfunction and dementia: what we need to know and do. *British Journal of Anaesthesia*.

[2] Monk TG, Weldon BC, Garvan CW, Dede DE, van der Aa MT, Heilman KM (2008). Predictors of cognitive dysfunction after major noncardiac surgery. *Anesthesiology*.

[3] Wang Y, Ma B, Wang C, Wang Y, Liu A, Hang L (2024). The influence of low-dose s-ketamine on postoperative delirium and cognitive function in older adults undergoing thoracic surgery. *Journal of Cardiothoracic Surgery*.

[4] Garutti I, Rancan L, Abubakra S, Simón C, Paredes SD, Ortega J (2019). Effects of Intraoperative Infusion of Esmolol on Systemic and Pulmonary Inflammation in a Porcine Experimental Model of Lung Resection Surgery. *Anesthesia and Analgesia*.

[5] Fu Z, Zhang J (2022). Molecular hydrogen is a promising therapeutic agent for pulmonary disease. *Journal of Zhejiang University. Science. B*.

[6] Yu X, Xiao H, Liu Y, Dong Z, Meng X, Wang F (2025). The Lung-Brain Axis in Chronic Obstructive Pulmonary Disease-Associated Neurocognitive Dysfunction: Mechanistic Insights and Potential Therapeutic Options. *International Journal of Biological Sciences*.

[7] Hosang L, Canals RC, van der Flier FJ, Hollensteiner J, Daniel R, Flügel A (2022). The lung microbiome regulates brain autoimmunity. *Nature*.

[8] He J, Zhang Y, Guo Y, Guo J, Chen X, Xu S (2024). Blood-derived factors to brain communication in brain diseases. *Science Bulletin*.

[9] Luo T, Deng Z, Ren Q, Mu F, Zhang Y, Wang H (2024). Effects of esketamine on postoperative negative emotions and early cognitive disorders in patients undergoing non-cardiac thoracic surgery: A randomized controlled trial. *Journal of Clinical Anesthesia*.

[10] McIntyre RS, Rosenblat JD, Nemeroff CB, Sanacora G, Murrough JW, Berk M (2021). Synthesizing the Evidence for Ketamine and Esketamine in Treatment-Resistant Depression: An International Expert Opinion on the Available Evidence and Implementation. *The American Journal of Psychiatry*.

[11] MacQueen GM, Memedovich KA (2017). Cognitive dysfunction in major depression and bipolar disorder: Assessment and treatment options. *Psychiatry and Clinical Neurosciences*.

[12] Höflich A, Kraus C, Pfeiffer RM, Seiger R, Rujescu D, Zarate CA (2021). Translating the immediate effects of S-Ketamine using hippocampal subfield analysis in healthy subjects-results of a randomized controlled trial. *Translational Psychiatry*.

[13] Wen Y, Xu J, Shen J, Tang Z, Li S, Zhang Q (2024). Esketamine Prevents Postoperative Emotional and Cognitive Dysfunction by Suppressing Microglial M1 Polarization and Regulating the BDNF-TrkB Pathway in Ageing Rats with Preoperative Sleep Disturbance. *Molecular Neurobiology*.

[14] Yuan J, Chen S, Xie Y, Wang Z, Xing F, Mao Y (2022). Intraoperative Intravenous Infusion of Esmketamine Has Opioid-Sparing Effect and Improves the Quality of Recovery in Patients Undergoing Thoracic Surgery: A Randomized, Double-Blind, Placebo-Controlled Clinical Trial. *Pain Physician*.

[15] Fan Q, Luo J, Zhou Q, Zhang Y, Zhang X, Li J (2023). Esketamine opioid-free intravenous anesthesia versus opioid intravenous anesthesia in spontaneous ventilation video-assisted thoracic surgery: a randomized controlled trial. *Frontiers in Oncology*.

[16] Yim AP, Wan S, Lee TW, Arifi AA (2000). VATS lobectomy reduces cytokine responses compared with conventional surgery. *The Annals of Thoracic Surgery*.

[17] Alam A, Hana Z, Jin Z, Suen KC, Ma D (2018). Surgery, neuroinflammation and cognitive impairment. *EBioMedicine*.

[18] Chen X, Liu Q, Fan L (2022). Effects of thoracic paravertebral block combined with s-ketamine on postoperative pain and cognitive function after thoracoscopic surgery. *Heliyon*.

[19] Wei X, Xing F, Xu Y, Zhang F, Cheng D, Zhou Y (2024). Preoperative gut microbiota of POCD patients induces pre- and postoperative cognitive impairment and systemic inflammation in rats. *Journal of Neuroinflammation*.

[20] Gao S, Dai H, Hao Q, Song J, Ji K, Xu H (2025). Effect of perioperative probiotic intervention on postoperative cognitive dysfunction in elderly patients: a randomized double- blinded and placebo-controlled trial. *Journal of Translational Medicine*.

[21] Zhu Y, Feng W, Kong Q, Sheng F, Li Z, Xu W (2023). Evaluating the effects of S-ketamine on postoperative delirium in elderly patients following total hip or knee arthroplasty under intraspinal anesthesia: a single-center randomized, double-blind, placebo-controlled, pragmatic study protocol. *Frontiers in Aging Neuroscience*.

[22] Marcantonio ER, Ngo LH, O’Connor M, Jones RN, Crane PK, Metzger ED (2014). 3D-CAM: derivation and validation of a 3-minute diagnostic interview for CAM-defined delirium: a cross-sectional diagnostic test study. *Annals of Internal Medicine*.

[23] Shen J, Song C, Lu X, Wen Y, Song S, Yu J (2023). The effect of low-dose esketamine on pain and post-partum depression after cesarean section: A prospective, randomized, double-blind clinical trial. *Frontiers in Psychiatry*.

[24] Hsiao WJ, Chen CY, Kang YN, Hu CJ, Chen CH, Lin PL (2023). Apolipoprotein E4 allele is genetically associated with risk of the short- and medium-term postoperative cognitive dysfunction: A meta-analysis and trial sequential analysis. *PloS One*.

[25] Arefayne NR, Berhe YW, van Zundert AA (2023). Incidence and Factors Related to Prolonged Postoperative Cognitive Decline (POCD) in Elderly Patients Following Surgery and Anaesthesia: A Systematic Review. *Journal of Multidisciplinary Healthcare*.

[26] Robinson TN, Raeburn CD, Tran ZV, Angles EM, Brenner LA, Moss M (2009). Postoperative delirium in the elderly: risk factors and outcomes. *Annals of Surgery*.

[27] Lechowicz K, Szylińska A, Listewnik M, Drożdżal S, Tomska N, Rotter I (2021). Cardiac Delirium Index for Predicting the Occurrence of Postoperative Delirium in Adult Patients After Coronary Artery Bypass Grafting. *Clinical Interventions in Aging*.

[28] Treccani G, Ardalan M, Chen F, Musazzi L, Popoli M, Wegener G (2019). S-Ketamine Reverses Hippocampal Dendritic Spine Deficits in Flinders Sensitive Line Rats Within 1 h of Administration. *Molecular Neurobiology*.

[29] Palotás A, Reis HJ, Bogáts G, Babik B, Racsmány M, Engvau L (2010). Coronary artery bypass surgery provokes Alzheimer’s disease-like changes in the cerebrospinal fluid. *Journal of Alzheimer’s Disease: JAD*.

[30] Zhou Y, Ma Y, Yu C, Chen Y, Ding J, Yu J (2022). Detection Analysis of Perioperative Plasma and CSF Reveals Risk Biomarkers of Postoperative Delirium of Parkinson’s Disease Patients Undergoing Deep Brain Stimulation of the Subthalamic Nuclei. *Clinical Interventions in Aging*.

[31] Baldeiras I, Santana I, Leitão MJ, Gens H, Pascoal R, Tábuas-Pereira M (2018). Addition of the Aβ42/40 ratio to the cerebrospinal fluid biomarker profile increases the predictive value for underlying Alzheimer’s disease dementia in mild cognitive impairment. *Alzheimer’s Research & Therapy*.

[32] Barthélemy NR, Salvadó G, Schindler SE, He Y, Janelidze S, Collij LE (2024). Highly accurate blood test for Alzheimer’s disease is similar or superior to clinical cerebrospinal fluid tests. *Nature Medicine*.

[33] Sun Y, Feng H, Zou T, Hou M, Jin Y, Gu C (2021). Assessment of risk factors for postoperative cognitive dysfunction after coronary artery bypass surgery: a single-center retrospective cohort study. *Bioscience Reports*.

[34] Butenschoen VM, Wriedt F, Meyer B, Krieg SM (2023). Neurocognitive monitoring in patients undergoing opioid pain medication after spinal surgery: a feasibility study of a new monitoring method. *Acta Neurochirurgica*.

[35] Fedgchin M, Trivedi M, Daly EJ, Melkote R, Lane R, Lim P (2019). Efficacy and Safety of Fixed-Dose Esketamine Nasal Spray Combined With a New Oral Antidepressant in Treatment-Resistant Depression: Results of a Randomized, Double-Blind, Active-Controlled Study (TRANSFORM-1). *The International Journal of Neuropsychopharmacology*.

